# The Complexities in Genotyping of Congenital Adrenal Hyperplasia: 21-Hydroxylase Deficiency

**DOI:** 10.3389/fendo.2019.00432

**Published:** 2019-07-04

**Authors:** Duarte Pignatelli, Berta L. Carvalho, Aida Palmeiro, Alberto Barros, Susana G. Guerreiro, Djuro Macut

**Affiliations:** ^1^Hospital S. João, Porto, Portugal; ^2^Department of Biomedicine, Faculty of Medicine of Porto, Porto, Portugal; ^3^IPATIMUP/I3S Research Institute, University of Porto, Porto, Portugal; ^4^Genetics, Department of Pathology, Faculty of Medicine, University of Porto, Porto, Portugal; ^5^I3S Research Institute, University of Porto, Porto, Portugal; ^6^Clinical Genetics Center, Porto, Portugal; ^7^Clinic of Endocrinology, Diabetes and Metabolic Diseases, Faculty of Medicine, University of Belgrade, Belgrade, Serbia

**Keywords:** 21OH deficiency, CAH—congenital adrenal hyperplasia, adrenal cortex, androgen excess syndromes, genotyping, endocrine genetics, rare diseases, disorders of sex development

## Abstract

The deficiency of 21-hydroxylase due to *CYP21A2* pathogenic variants is a rather frequent disease with serious consequences, going from a real mortality risk to infertility and to milder symptoms, nevertheless important for affecting the patients' self-esteem. In the most severe cases life-threatening adrenal salt wasting crises may occur. Significant morbidity including the possibility of mistaken gender determination, precocious puberty, infertility and growth arrest with consequent short stature may also affect these patients. In the less severe cases milder symptoms like hirsutism will likely affect the image of the self with strong psychological consequences. Its diagnosis is confirmed by 17OH-progesterone dosages exceeding the cut-off value of 10/15 ng/ml but genotyping is progressively assuming an essential role in the study of these patients particularly in confirming difficult cases, determining some aspects of the prognosis and allowing a correct genetic counseling. Genotyping is a difficult process due to the occurrence of both a gene and a highly homologous pseudo gene. However, new tools are opening new possibilities to this analysis and improving the chances of a correct diagnosis and better understanding of the underlying mechanisms of the disease. Beyond the 10 classic pathogenic variants usually searched for in most laboratories, a correct analysis of 21OH-deficiency cases implies completely sequencing of the entire gene and the determination of gene duplications. These are now recognized to occur frequently and can be responsible for some false positive cases. And finally, because gene conversions can include several pathogenic variants one cannot be certain of identifying that both alleles are affected without studying parental DNA samples. A complete genotype characterization should be considered essential in the preparation for pregnancy, even in the case of parents with milder forms of the disease, or even just carriers, since it has been reported that giving birth to progeny with the severe classic forms occurs with a much higher frequency than expected.

## Introduction

The congenital adrenal hyperplasias (CAH) are a group of autosomal recessive disorders that are caused by decreased activity of one of the enzymes involved in the steroidogenic pathway of the adrenal cortex, leading to impaired synthesis of cortisol by the adrenal gland. The vast majority of the cases of CAH (95%) are due to 21-hydroxylase deficiency and associated with pathogenic variants in the 21-hydroxylase (*CYP21A2*) gene. This form of CAH will be the major focus of this article. Most affected individuals are compound heterozygotes, presenting different pathogenic variants on each allele rather than being homozygous for the same pathogenic variant. Although there seem to be some exceptions, most heterozygotes/carriers are asymptomatic.

Complete loss of function pathogenic variants of the CYP21A2 gene are associated with impaired cortisol and aldosterone synthesis. The accumulation of steroid precursor molecules leads to increased adrenal androgen production utilizing the delta-5 pathway and CYP17A1. Decreased cortisol concentrations result in loss of the negative feedback inhibition leading to a compensatory increase of adrenocorticotropic secretion (ACTH) and hypertrophy of the adrenal cortex.

The clinical importance of CAH results from the possible occurrence of adrenal insufficiency, genital ambiguity, short stature, androgen excess syndromes and infertility.

With increased awareness of the signs and symptoms of CAH, morbidity and mortality has decreased. Hormone replacement therapy is beneficial, but affected individuals require very precise and personalized treatment regimens for optimal outcomes.

Thus, clinicians need to be aware of the potential consequences and complications of CAH. Specific issues include concerns regarding genital ambiguity in affected females, premature pubarche, accelerated skeletal maturation with reduced final height, bone health, adrenal tumors, and testicular adrenal rests tumors (TARTs). Although more common among affected females, infertility can affect both genders. Genetic counseling is essential, especially since this disease affects many individuals at reproductive age (1–3).

In spite of the fact that this disorder results from a continuum of enzymatic deficiencies, congenital adrenal hyperplasia has been classified into three main forms (Table 1).

**Table 1:** Phenotypes of 21-hydroxylase deficiency.

**Classic Salt-Wasting—very severe (0% enzymatic activity)**
Failure to thrive
Cortisol deficiency
Mineralocorticoid deficiency
Hyponatremia
Hyperkalemia
High PRA
Hypovolemic shock
Excess androgen production, early in life
Virilization of external genitalia in females
**Classic Simple Virilizing—intermediate severity (1–2% enzymatic activity)**
Virilization of external genitalia in females
Progressive Premature Pubarche
Progressive virilization with clitoromegaly (female) or increased penile size (male)
Elevated androgen levels cause accelerated growth velocity and advanced bone age but premature fusion of the epiphyses is also observed causing final short stature.
**Non-Classic Adrenal Hyperplasia—milder form (20–50% enzymatic activity)**
Between asymptomatic or with signs of androgen excess appearing later in life (acne; hirsutism; menstrual irregularities; anovulation; infertility).

The salt-wasting or salt-losing form is associated with complete loss of 21-hydroxylase activity leading to deficient cortisol and aldosterone biosynthesis.

Prior to newborn screening programs, affected females were more rapidly identified due to the simultaneous presence of genital ambiguity. The genital ambiguity involves enlargement of the phallus, varying degrees of fusion of the labioscrotal folds, and non-palpable gonads. Affected males typically presented within the first 2 weeks of life with failure to thrive, vomiting, hypotension, hyponatremia, and hyperkalemia.

The simple virilizing form often presents with genital ambiguity without overt salt loss in affected females. This simple virilizing form may present with phallic enlargement, premature development of sexual hair, and initially tall stature, accompanied by advanced skeletal maturation resulting in final short stature. Children with simple virilizing CAH generally synthesize sufficient aldosterone and so they are not overt salt-losers.

Salt-losing and simple virilizing CAH are often grouped together as the classic forms. The incidence of classic CAH is reported as being of 1:15,000 live births. Consequently, the carrier frequency is ~1:60 (4–9). In black Americans the incidence is much lower, going from 1:25,000 to 1:42,000 in different studies (10, 11).

The most common form of CAH is the non-classic or late onset form (NCAH). The characteristic features of NCAH, hirsutism, irregular menses, and infertility, lead to an ascertainment bias favoring diagnosis of affected females. Affected males are usually only identified through family studies. Overt glucocorticoid and mineralocorticoid deficiency are unusual. Although patients with NCAH usually have no evidence of ACTH or CRH excess, some may demonstrate an increased glucocorticoid response to ACTH stimulation, possibly reflective of subtle adrenal hyperplasia (12–14). The reason for the existence of increased androgen production by the adrenals without an increase in ACTH has been attributed to an altered enzymatic kinetic of *CYP21A2* (15). The elevated androgen levels in NCAH may also result from ovarian hypersecretion since the ovaries in NCAH women are frequently polycystic (15, 16), and from peripheral conversion of precursors.

NCAH affects between 0.1 and 1% of the general population. Among hirsute women its prevalence reaches between 1 and 10% (16–20). The most recent meta-analysis indicated the prevalence of 4.2% among women with androgen excess worldwide (21). One clinical study based on ACTH-stimulated 17-OHP concentrations reported the incidence to be highest among Ashkenazi Jewish populations (22).

The signs and symptoms of NCAH are similar to those of Polycystic Ovary Syndrome (PCOS) (16). Since the treatment, potential complications, and genetic implications differ between these 2 syndromes, accurate diagnosis is important and that may impose a complete differential diagnosis in every case of hirsutism and a surveillance of metabolic dysfunction (e.g., insulin resistance) and subsequent prevention of the increased cardiovascular risk not only in PCOS but also in NCAH cases (16).

## Molecular Genetic Testing

### *CYP21* Gene Structure

**Figure 1:**
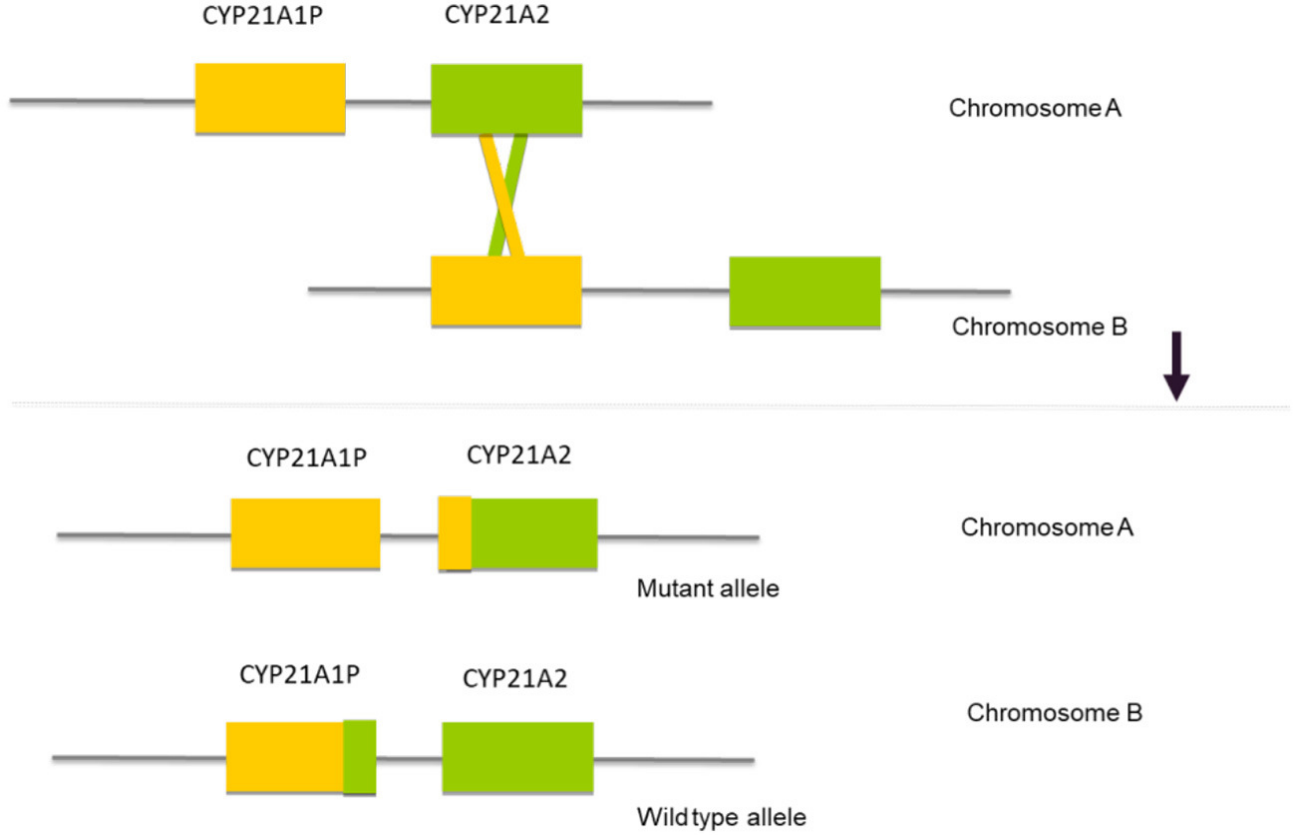
Schematic representation of the mechanism of gene conversion, where a misalignment between the two DNA sequences results in a recombination between the *CYP21A2* gene and the *CYP21A1P* pseudogene.

The *CYP21A2* gene is located in the long arm of chromosome 6, within the major human histocompatibility complex (HLA), a region that displays a complex organization of genes with a great variability in gene size and copy numbers (2, 3, 23, 24). Approximately 30 kb from the *CYP21A2* gene there is a non-functional pseudogene—*CYP21A1P*. Both, the functional gene and the pseudogene comprise ten exons and share a high level of nucleotide sequence identity of 98% in their exons and 96% in their introns (25, 26). The pseudogene *CYP21A1P* is inactive because of the presence of multiple pathogenic variants, small insertions or deletions and point pathogenic variants that prevent the synthesis of a functional protein. The location and high rate of homology between the two genes facilitates misalignment that results in recombination events between the gene and the pseudogene (Figure 1). These events that are called gene conversions constitute a mutagenesis mechanism that contributes to the majority of the point pathogenic variants in the *CYP21A2* gene.

**Figure 2:**
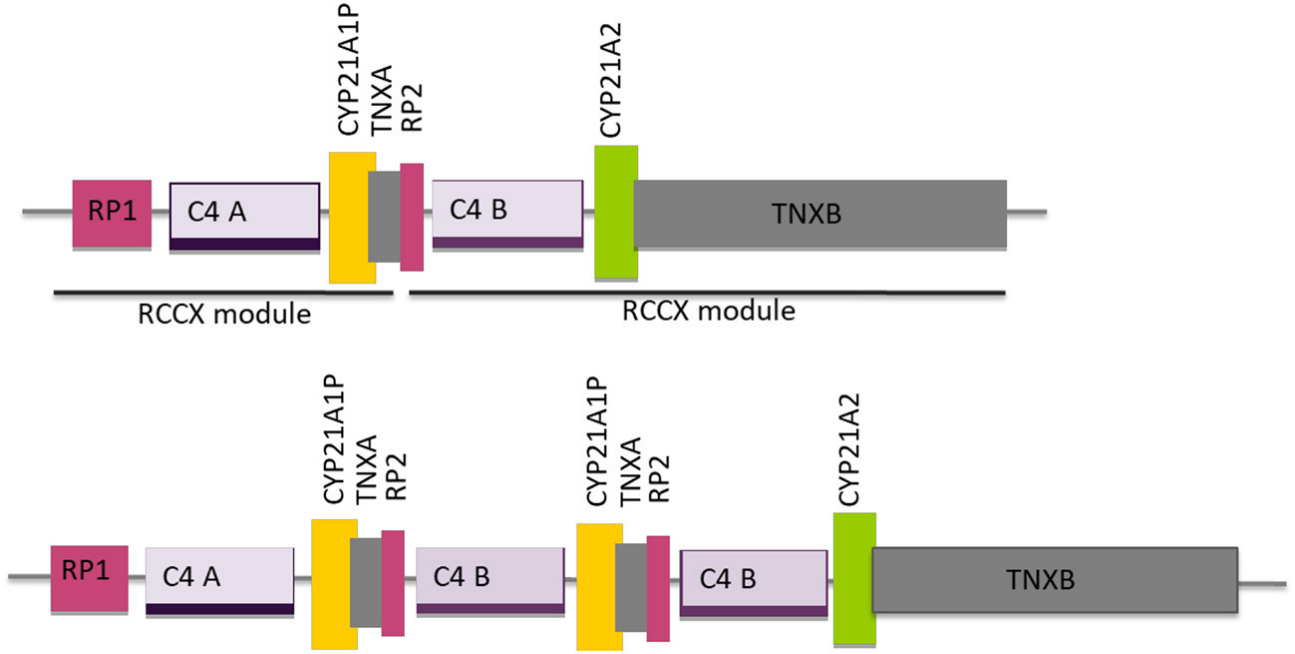
Schematic diagram of the organization of the *RCCX* modules, one with the *CYP21A1P* pseudogene and the other with the *CYP21A2* gene for the most common bimodular haplotype and for the three modular haplotype with two modules harboring the *CYP21A1P* pseudogene and one the *CYP21A2* gene. Adapted from Sweeten et al. (27).

Neighboring the *CYP21A2* and the *CYP21A1P* genes there are three other genes, *RP1, C4, TNXB* and two truncated pseudogenes, *RP2* and *TNXA*, that together, constitute a genetic unit designated RCCX module (RP-C4-CYP21-TNX) (Figure 2) and correspond to a highly variable stretch of DNA of ~30 Kb (28). The genes *C4B* and *C4A* encode for the fourth component of serum complement (29, 30) the gene *TNXB* for an extracellular matrix protein termed tenascin-X^23^ and the *RP1* gene for a serine/threonine nuclear protein kinase (28). The usual organization is bimodular, and consists of two RCCX modules, one with the *CYP21A1P* pseudogene and the other with the *CYP21A2* gene, where the orientation of the genes from telomere to centromere is: *RP1*-*C4A*-*CYP21A1P*-*TNXA*-*RP2*-*C4B*-*CYP21A2*-*TNXB*. This bimodular haplotype is present in about 69% of the Caucasian population, while a monomodular haplotype occurs with a frequency of 17% and a “three modular” haplotype in about 14% of the cases (Figure 2) (28, 31). The majority of the trimodular haplotypes carry two copies of the *CYP21A1P* pseudogene and one copy of the *CYP21A2* gene, but the haplotype with two copies of the *CYP21A2* gene and one copy of the *CYP21A1P* pseudogene is also possible and has been described in carriers of the p.(Gln319^*^) pathogenic variant and of chimeric *CYP21A1P*/*CYP21A2* genes (31–34).

The frequent existence of copy number variations together with the large number of possible genetic variants makes the characterization of *CYP21A2* alleles rather difficult. Pathogenic variants have been identified both in the coding and non-coding regions of the gene inclusively in the 5′UTR and the 3′UTR regions. Consequently, it is important to screen all coding exons, as well as intron-exon boundaries of the gene.

### *CYP21A2* Pathogenic Alterations

Due to gene and pseudogene location and the highly polymorphic complexity of the region, recombination events are the major cause of *CYP21A2* pathogenic variants.

Intergenic recombinations are responsible for more than 95% of the pathogenic variants causing 21OHD. Approximately 75% of the deleterious variants are transferred by small conversions from the pseudogene during meiosis. These conversions can involve one or more pseudogene variants. They are called “microconversions,” when they are limited to a single point variant.

In the remaining 20–25% of the cases, CAH is due to gross misalignment owing to unequal crossing over during meiosis that can lead to gene deletions, gene duplications and deletions involving *CYP21A2* and other contiguous genes (35, 36). CAH can also be caused by uniparental isodisomy but this is rare (37).

To date more than 1,300 genetic variants have been reported but only 230 affecting human health (38). The majority of these pathogenic variants result in classic form cases (156 in the total 230) (38). Nineteen genetic variants have been described in the non-translated regions of the gene. Of these, 4 affect the promoter, resulting in promoter conversion: c.(-126C>T; −113G>A; −110T>C; and−103A>G). c-126C>T was reported to cause NCAH (39).

One hundred and fifty three of the 230 genetic variants were demonstrated to be missense mutations (38). These can result in all forms of the disease while nonsense and frameshift mutations always result in the classic forms.

**Figure 3:**
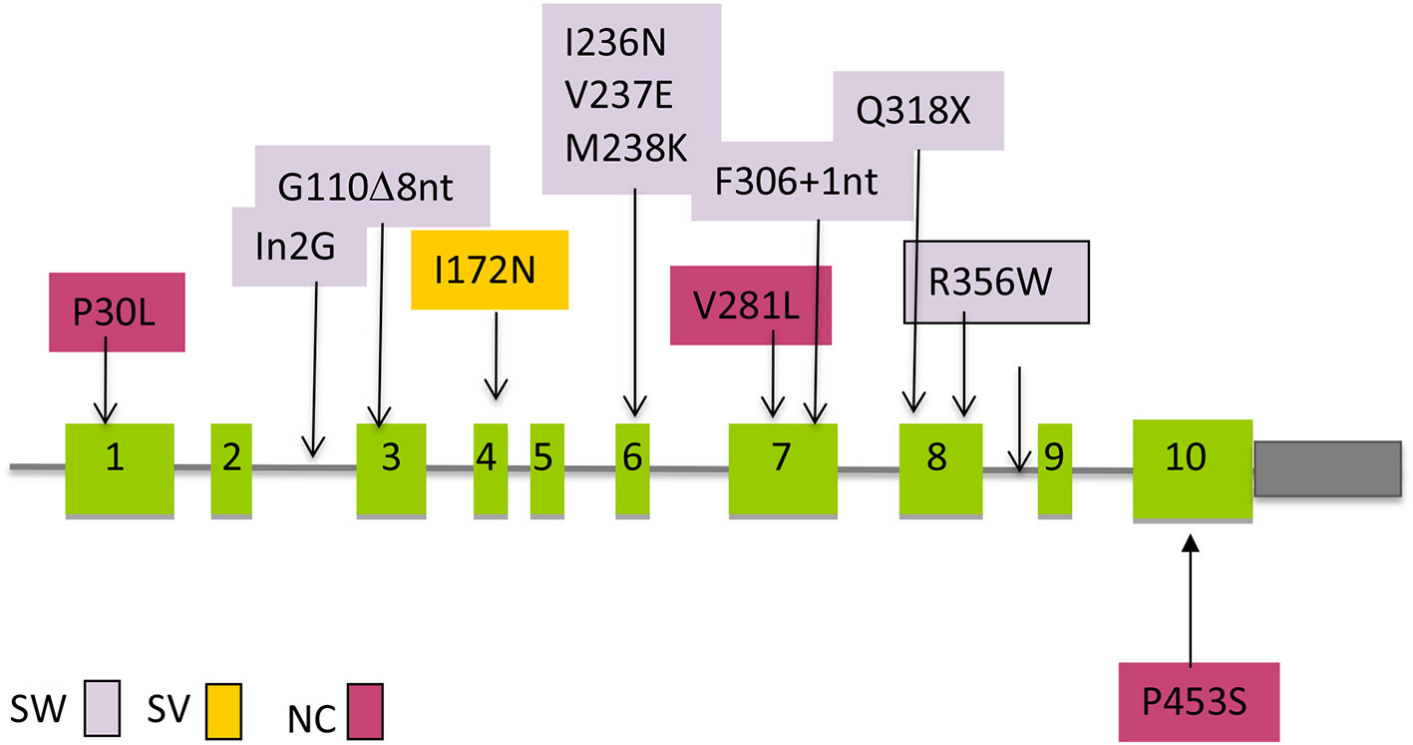
Distribution of the most common mutations along the *CYP21A2* gene that are transferred by gene conversion and the association with clinical severity. SW, salt wasting; SV, simple virilizing; NC, Non-classic.

The real life situation, however, can be much simplified as there is a small group of pathogenic variants that account for the great majority of affected alleles (*n* = 10) (Figure 3). The screening strategy to search for those most common pathogenic variants is an usual practice among molecular geneticists as the process is less expensive and less time consuming.

Whenever possible familial segregation studies should be done, in which both parents are studied together with the proband, so that one may know if two detected pathogenic variants affect the 2 alleles (*trans* configuration) or are located in the same allele (*cis* configuration). In this last situation there is only one allele with mutations and the other allele is normal. That person will not be clinically affected in spite of having 2 pathogenic variants on the CYP21A2 gene.

Molecular genetic testing of the CYP21A2 gene should be considered essential since it allows the establishment of correlations between genotype and phenotype, confirming the clinical and biochemical diagnosis, inferring about the severity status of the patients, distinguishing between severe and milder cases and, very importantly, allowing a correct genetic counseling for any couple at risk and their relatives.

### CYP21A2 Genetic Variants

Two types of recombination can be considered: one is the result of an unequal crossing over during meiosis, with the production of large rearrangements and the other consists of smaller gene conversions where a segment of the functional *CYP21A2* gene is replaced by a segment copied from the *CYP21A1P* pseudogene (Figure 1). The segment of the converted *CYP21A2* gene will carry either a few nucleotides from CYP21A1P (microconversions) or a short sequence affecting one or more exons (25, 40–42). The converted sequences harbor pathologic variants so that they will inactivate or at least significantly modify the normal *CYP21A2* gene translation of the protein.

#### Large Deletions and Gene Conversions

**Figure 4:**
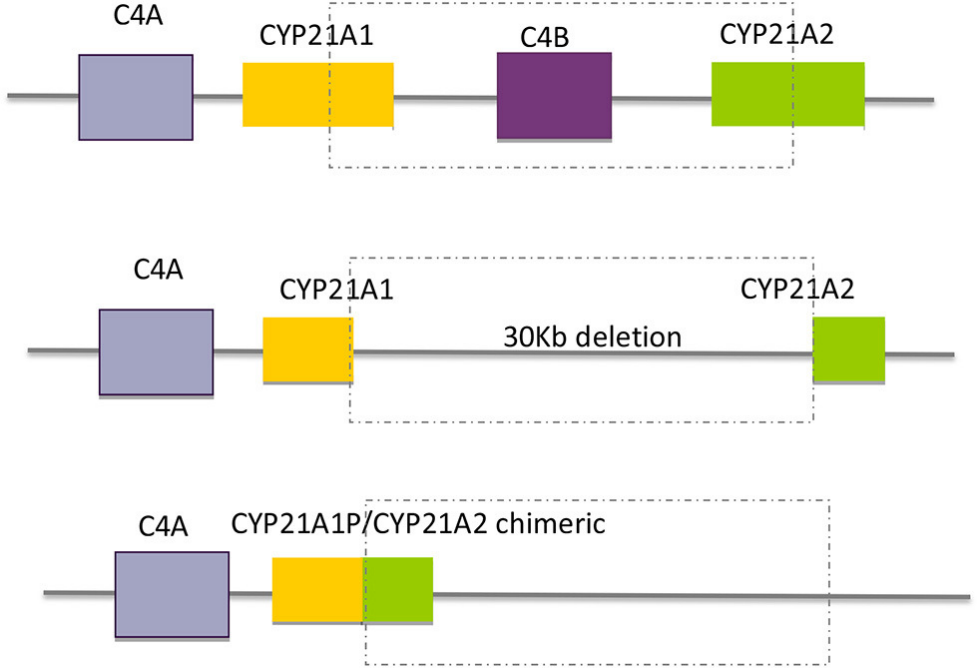
Schematic diagram of the formation of chimeric genes by large gene deletions.

Large gene conversions and large deletions, sometimes involving C*4B* and *CYP21A2* with the formation of *CYP21A1P*/*CYP21A2* chimeric genes comprise ~20% of the pathogenic variants. In the last situation a 26 or 32 Kb deletion (depending on whether *C4B* is the long or short gene), involving the 3′ end of *CYP21A1P*, all of the *C4B* gene, and the 5′ end of the *CYP21A2* gene, produces a single non-functional chimeric gene with its 5′ and 3′ ends corresponding to *CYP21A1P* and *CYP21A2*, respectively (Figure 4). Several different chimeric *CYP21A1P*/*CYP21A2* genes have been found and characterized (43–47).

#### Point Pathogenic Variants and Small Deletions/Insertions

Approximately 75% of the intergenic recombinations correspond to pathogenic variants normally present in the pseudogene that are transferred to the functional gene by microconversion events (Figure 3) (41). Other rearrangements, such as a deletion of 10 nucleotides in exon 8 and a duplication of 16 nucleotides in exon 9 have also been reported.

#### P30L: Pro-30Leu (p.(Pro31Leu))

This pathogenic variant yields an enzyme with 20–60% of normal activity when expressed in cultured cells (48). However, enzymatic activity is rapidly lost when the cells are lysed, suggesting that the enzyme is relatively unstable. Patients carrying this pathogenic variant tend to have more severe signs of androgen excess than patients carrying the more common non-classic pathogenic variant V281L (p.(Val282Leu) (48, 49). This pathogenic variant is found in approximately one-sixth of alleles in patients with non-classic disease, but it may comprise a higher percentage in Japan (50).

#### IVS2-13A/C>G: A or C-G Pathogenic Variant in Intron 2 (c.293-13A/C>G)

This pathogenic variant is characterized by the substitution of A or C nucleotide at 13 bp before the end of intron 2 (nt 656) to G. This pathogenic variant causes aberrant splicing of intron 2 with retention of 19 nucleotides normally spliced out of mRNA, resulting in a shift in the translational reading frame (51, 52).

#### G110Δ8nt (p.(Gly111Valfs^*^21))

This deletion of eight nucleotides (8-nt) in exon 3 prevents the synthesis of the protein by a frameshift and causes a salt-wasting type of CAH (40). It is present in about 8% of the salt-wasting cases.

#### I172N: Ile-172Asn (p.(Ile173Asn))

This pathogenic variant results in an enzyme with ~1% of normal activity (53, 54) and has been specifically associated with the simple virilizing form of the disease; however it has also been described in the salt wasting form (55).

#### Cluster in Exon 6: I236N/V237E/M238K: Ile-Val-Met-236–237-238-Asn-Glu-Lys

##### (p.(Ile237Asn), p.(Val238Glu), p.(Met239Lys))

This cluster of three missense pathogenic variants in the G helix also abolishes enzymatic activity possibly by interference with substrate binding (52, 54).

##### V281L: Val-281Leu (p.(Val282Leu))

This pathogenic variant results in an enzyme with 50% of normal activity when 17-OHP is the substrate but only 20% of normal activity for progesterone (54, 56). V281L occurs in the majority of patients with non-classic 21-hydroxylase deficiency who carry the HLA haplotype B14; DR1, an association that is consistent to a founder effect (57). Overall, ~70% of all non-classic alleles carry the V281L pathogenic variant (58, 59).

##### F306+T: L306insT (p.(Leu307Phefs^*^5))

This 1-nt insertion in exon 7 of *CYP21A1P* has generally been described not as an independent pathogenic variant but in a cluster of pseudogene derived pathogenic variants in exons 7 and 8 particularly in Dutch patients (60).

##### Q318X: Gln-318-Term (p.(Gln319^*^))

A nonsense pathogenic variant in codon 318 (Q318X) where the CAG, encoding glutamine changes to TAG, a nonsense codon that is predicted to result in a completely non-functional enzyme due to premature termination of translation (61).

##### R356W: Arg-356Trp (p.(Arg357Trp))

This pathogenic variant abolishes enzymatic activity when expressed in mammalian cells (52, 53). It is located in a region of the gene encoding the K helix of the enzyme, which suggests that the pathogenic variant affects interactions with the cytochrome P450 reductase (POR), but this has not been demonstrated experimentally (62).

##### P453S: Pro-453Ser (p.(Pro454Ser))

This missense pathogenic variant results from a transition of a CCC to a TCC and was initially described as not present in the pseudogene (63, 64). Although the functional mechanism is not clearly explained it corresponds to a decrease of 50–68% of 17-OHP and 20–46% of progesterone utilization (65). It occurs in a number of different populations and suggests that *CYP21A1P* may carry P453S as an occasional polymorphism and that this pathogenic variant is transferred to *CYP21*A2 in the same way as the other pathogenic variants frequently causing 21-hydroxylase deficiency (2).

#### Other Pathogenic Variants

More than 200 different pathogenic variants have been described and this number is increasing with the improvements of molecular diagnosis (see http://www.cypalleles.ki.se/cyp21.htm and http://www.hgmd.cf.ac.uk). Some of these pathogenic variants have been reported in several cases, but most of them are private family pathogenic variants, which means that they were described only in one family. Except for the nonsense, frameshift, and rearrangement alterations that are deduced as severe, most of these pathogenic variants are missense, and require functional studies to be classified.

Less than 5% of the pathogenic variants in the *CYP21A2* gene are not caused by gene conversions and possibly are not present in the pseudogene (66, 67).

In a study trying to establish a phenotype-genotype correlation of 13 rare *CYP21A2* pathogenic variants (68) it was demonstrated that some were associated with the severe SW form (L167P, G291S, G292D, and R354H), some with the SV form (I77T, E320K, R341P, and G424S) and with the NC form (I230T and R233K) but at the same time it was observed that some of these pathogenic variants conferred different phenotypes depending on if they were isolated or associated with another pathogenic variant. This was the case of the pathogenic variant I230T responsible for a NC form that if associated with the V281L pathogenic variant corresponded to a more severe phenotype. This synergistic effect that results in a different phenotype has also been described for other pathogenic variants, such as H62L (35), R339H (69), or P105L (65) with P453S.

#### Polymorphisms

Some variants do not affect the protein production and are considered normal polymorphisms (2). One of these variants, D183E is also present in the *CYP21A1P* gene and represents a gene conversion that does not affect the enzyme activity while others, like K102R, S268T, and N493S have been described only in the *CYP21A2* gene.

## Genotyping and Pregnancy

Genotyping of *CYP21A2* gene is strongly recommended particularly in couples that have the intention to conceive, both to confirm the diagnosis in difficult cases but mostly to be able to do a correct genetic counseling. Although the correlation between genotype and phenotype is high, sometimes the interpretation of the genotypes is rather difficult as we will demonstrate.

The risk for a woman with CAH to have an infant with CAH depends on her partner's genotype. If her partner does not carry a mutant *CYP21A2* allele, all of her children will be carriers but will not have the disease. If the woman is homozygous for a mild pathogenic variant, such as V281L (p.(Val282Leu)) and her partner carries a *CYP21A2* pathogenic variant, the probability is that 50% of her children will have NCAH. Since the probability of a person in the general population being a carrier for a severe pathogenic variant is 1.7% (1 in 60) (22) and the probability of a patient with NCAH having a severe pathogenic variant together with a mild one is ~60% (as this occurs in 2/3 of the cases) (70), the risk for having a child with classic CAH would be expected to be 1:600. However, it was demonstrated that the real frequency is closer to 2.5% (71) and this increased risk was attributed to presumably higher carrier frequencies in certain ethnic groups. Thus, the genotyping of both parents should be a component of the pre-natal study protocol for families in which one parent has CAH (71).

## Genotype-Phenotype Correlations

In general terms there is a good correlation between genotype and phenotype (90–95%).

Some pathogenic variants translate into the most severe forms of the disease (enzymatic deficiency of almost 100% or, in other terms no 21-hydroxylase activity) resulting in the salt-wasting forms of CAH. These pathogenic variants are called Severe pathogenic variants (Table 2).

**Table 2:** Genotype-phenotype correlation for the most common pathogenic variants, according to the percentage of enzyme activity.

	**Variant**	**% Enzyme active**	**Phenotype**
Severe	Gene deletions and Large gene conversions	0%	Classic—Salt wasting CAH
	8bp del		
	E6 cluster		
	Q318X (p.(Gln319*))		
	R356W (p.(Arg357Trp)		
Intermediate	I172N (p.(Ile 173 Asn))	1–2%	Classic—Simple virilizing CAH
Mild	P30L(p.(Pro31Leu))	20–60%	Non-classic CAH
	P453S (p.(Pro454Ser)		
	R339H (p.(Arg340His))		
	R369W (p.(Arg370Trp)		
	I230T (p.(Ile231Thr))		
	V281L (p.(Val282Leu))		

Missense pathogenic variant I172N (p.(Ile173Asn)) confers around 1–2% 21-hydroxylase activity. This results in a near normal aldosterone synthesis and so it is associated with the simple virilizing form of CAH. These are called the Intermediate pathogenic variants (Table 2).

A third group of pathogenic variants including P30L (p.(Pro31Leu)), P453S (p.(Pro454Ser), R339H (p.(Arg340His)), R369W (p.(Arg370Trp)), I230T (p.(Ile231Thr)) (68), and V281L (p.(Val282Leu)) (clearly the most frequent pathogenic variant in NCAH cases in every series) result in a more substantial preservation of enzymatic activity (20–60%) and are associated with the NCAH forms (Mild pathogenic variants) (Table 2).

Since most of 21-hydroxylase deficient CAH patients are compound heterozygotes:

(1) The most severe phenotypes (the classic forms) must have two severe pathogenic variants and no mild pathogenic variants

(2) NCAH patients may have two mild pathogenic variants (25–50%) or one mild and one more severe pathogenic variant (50–75%). The mild pathogenic variant allows the synthesis of 21-hydroxylase up to 50% of the normal activity in spite of the fact that the severe pathogenic variant would not contribute to any synthesis.

These pathogenic variants are substantially correlated with the clinical severity and with the different clinical forms of disease—salt-wasting, simple virilising, and non-classical. This is particularly true in patients with severe pathogenic variants. A greater diversity of clinical phenotypes can be observed in patients with less severe pathogenic variants where although the phenotype can be predicted to correspond to the less severely affected allele, the presence of a second allele with a severe or an intermediate pathogenic variant, can result in a more severe clinical phenotype (65, 68, 69, 72, 73).

It was also reported that NCAH patients with a mild plus a severe pathogenic variant had more intense degrees of hirsutism and higher 17OHP levels both basal and after ACTH stimulation than those with two mild pathogenic variants (70, 74).

Heterozygotes, also, have 17OH-progesterone responses to ACTH stimulation that are clearly above normal even though not attaining the levels of patients bearing the disease. These results await further developments.

There are several examples that, in spite of the general assumption that there is a relatively high concordance between genotype and phenotype there is some variability, particularly in the moderately affected patients (75–77). The pathogenic variants designated as *IVS2-13* (c.293-13A/C>G) and *I172N* (p.(Ile173Asn)) for instance result in variable degrees of 21-hydroxylase activity (possibly through alternative splicing) hence explaining that patients that would generally be expected to be Simple Virilizing cases may sometimes be Salt-Wasting and others also be closer to NCAH (51, 60). Another example is the P30L (p.(Pro31Leu)) pathogenic variant which can be associated with cases of NCAH as well as cases of SV-CAH (64).

An explanation for some lack of correlation between genotype and phenotype may result from not sequencing the whole gene in most studies hence not having a full picture of the total number of pathogenic variants.

In conclusion, the clinical condition related to 21-hydroxylase deficiency represents a continuum of reductions in enzyme activity of which the 3 levels of severity generally considered, represent merely a systematization to guide and facilitate the clinical practice (15). Finally, it was also recognized that the phenotype can change with time which implies the impossibility of a perfect correlation between genotype and phenotype.

## Prevalence in Different Ethnic Populations

Reports regarding the incidence and percentage of specific pathogenic variants among different ethnic groups have been published (78). The V281L (p.(Pro282Leu)) pathogenic variant is the most common in Ashkenazi Jews (allelic frequency of 63%). Large deletions are frequent in Anglo-Saxons (28%). The Q318X (p.(Gln319^*^)) was found in 16% of the East Indians. In Croatians, the R356W (p.Arg356Trp)) pathogenic variant was the most frequent (14%).

V281L (p.(Val282Leu), the most common pathogenic variant in most of the European populations, was not detected in Yupik-speaking, Eskimos of Western Alaska, Native Americans, East Indians and Asians. The Yupik Eskimos, representing an isolated geographic population with founder effect, carry the IVS2-13A/C>G: A or C-G pathogenic variant in intron 2 (c.293-13A/C>G).

In a study of a large French population (68) the frequency of the most common pathogenic variants was, for the classical form: int2 (c.293-13A/C>G) (30%), large rearrangements (25%), I172N (p.(Ile173Asn)) (17%), and Q318X (p.(Gln319^*^)) (7%) and for the NC form V281L (p.(Val282Leu) (55%), int2 (c.293-13A/C>G) (9%) large rearrangements (8%), I172N (p.(Ile173Asn)) (4%), and Q318X (p.(Gln319^*^)) (3%).

Three novel pathogenic variants, an insertion 1,003^∧^1,004 insA in exon 4, a C>T transition in codon 408 (p.(Arg408Cys)) and a A>G transition in the intron IVS2-2A>G were described in Brazil and suggested to be due to a founder effect, as was previously found for another pathogenic variant (G424S) in the same population (79, 80).

In Finland, there seem to be multiple independent founder *CYP21A2* gene pathogenic variants, each one associated with a different haplotype, where some are identical to those observed in other Europe populations, probably introduced by immigrants from Scandinavian or Baltic origin during the first centuries AD and others found only locally and with a more recent origin. The study of this diversity provided important informations about migrations between and within populations (81, 82).

In Tunisia, there is a high prevalence of the pathogenic variant Q318X (p.(Gln319^*^)) (35.8%) (83).

A study in Iran, on the contrary, demonstrated that the most frequent pathogenic variants in the CYP21 gene were in2G, del-CYP21A2, and I172N (p.(Ile 173 Asn)). Unlike in other ethnic groups, there was no R356W (p.(Arg357Trp)) pathogenic variant, and a higher rate of del-8bp (10%) was found (84).

In Lebanese, for the classical forms, the most frequent pathogenic variant was the splice site pathogenic variant in intron 2 accounting for 39% of the disease alleles, gene conversions accounted for 14% of the alleles, but no large deletions were found. In non-classical forms, the V281L (p.(Val282Leu)) pathogenic variant in exon 7 represented 86% of the tested alleles (85).

*De novo* deletions and *de novo* apparent conversions have been reported, comprising about 1% of 21-hydroxylase-deficient alleles. The allele frequency of de novo gene conversion in intron 2 in the general population is estimated 1 in 2 × 10^4^ (2).

Different chimeric *CYP21A1P*/*CYP21A2* genes have been described in different populations, some of them in Taiwanese (45, 46, 86), and the others in patients of Caucasian origin (43, 44, 47).

## Genetic Testing

PCR-based mutation-detection methods with sequencing of the entire gene and multiplex ligation-dependent probe amplification are nowadays the golden standard for genotyping the *CYP 21A2* gene.

### General Considerations

The specific gene amplification by PCR has dramatically improved the sensitivity of the different techniques to detect *CYP21A2* gene pathogenic variants. However, it was initially difficult to use PCR because of the paucity of primers that would amplify *CYP21A2* without amplifying the highly homologous *CYP21A1P* pseudogene, which carries most of the pathogenic variants of interest.

With time, however, PCR conditions were identified that permitted gene-specific amplification of *CYP21A2* in two segments.

PCR-based diagnosis may be complicated by failure to amplify one haplotype and result in misdiagnosis. Examination of flanking microsatellite markers in all family members can minimize this problem.

If only a DNA sample from the patient is analyzed, it is impossible to distinguish between compound heterozygosity for different pathogenic variants in *trans* and the presence of 2 pathogenic variants in the same allele allele (*cis*). Therefore, ideally both parents should also be analyzed so as to most reliably determine the phase of different pathogenic variants (i.e., whether they lie on the same or opposite alleles). Analysis of parental alleles also permits homozygotes and hemizygotes (i.e., individuals who have a pathogenic variant on one chromosome and a deletion on the other) to be distinguished.

In the approach of genetic testing for CAH caused by *CYP21A2* pathogenic variants we can consider three groups of studies:

1- Targeted analysis by screening of the most common *CYP21A2* pathogenic variants
2- Duplications and deletions
3- Whole gene sequencing.

## Targeted Pathogenic Variant Analysis

This approach is designed to detect the most common pathogenic variants described above. A number of different methods and strategies have been described that cover a variable range of pathogenic variants (87, 88).

### Allele-Specific Oligonucleotide Hybridization

**Figure 5:**
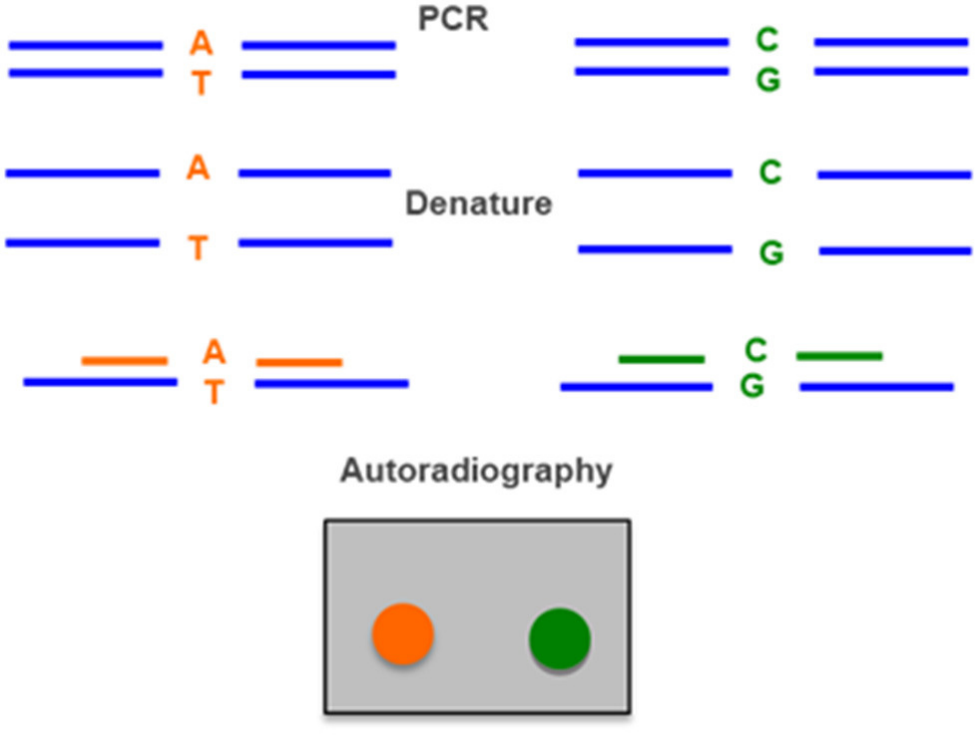
Allele-specific oligonucleotide hybridization (ASO) uses two radiolabeled probes, one for the wild type allele (A–T) and one for the allele with the mutation (C–G). DNA amplified by PCR is denatured, applied in a membrane and hybridized with the two probes. The result is detected by autoraradiography.

This method is based on the hybridization with allele-specific oligonucleotide (ASO) probes, which are short (typically 19–21 nucleotides) single-stranded DNA segments with the specific sequence of each polymorphic or mutant nucleotide in the gene. These probes are usually radioactively labeled. DNA amplified by PCR is dotted on filters and hybridized with the probes corresponding to the normal and mutant sequences for each of the frequently occurring gene conversions (Figure 5) (40, 63, 89).

### Amplification-Created Restriction Sites

**Figure 6:**
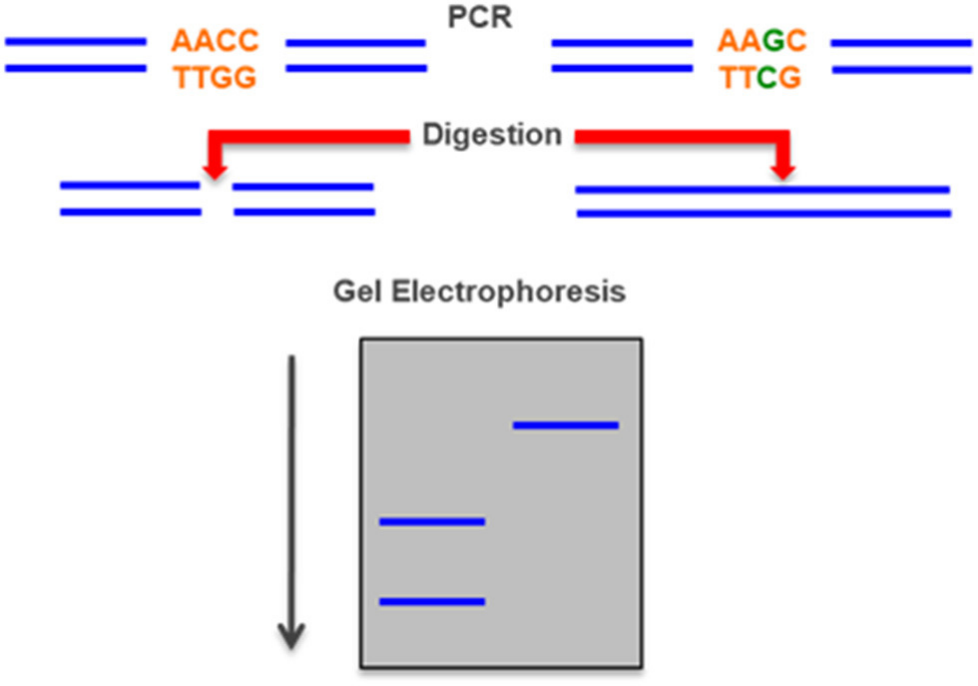
Restriction Fragment Length Polymorphism (RFLP)—a mutation can create or destroy a site that is digested by a specific a restriction enzyme. The mutation can be detected by the different length of a PCR-amplified DNA fragment between the normal and the mutant allele after separation in an electrophoresis gel.

Several pathogenic variants causing 21-hydroxylase deficiency (e.g., V281L and Q318X) create or destroy restriction sites and can thus be detected after digestion of a PCR-amplified DNA fragment with a restriction enzyme and subsequent analysis in agarose gels stained with ethidium bromide. If a restriction site does not exist it can be created by changing the sequence during the PCR with a modified primer and introduce a polymorphic restriction site into the amplified segment. This method thus involves a series of second round PCRs and several different restriction digests but does not require radioactivity or specialized equipment (Figure 6) (89, 90).

### Single-Stranded Conformation Polymorphisms (SSCP)

**Figure 7:**
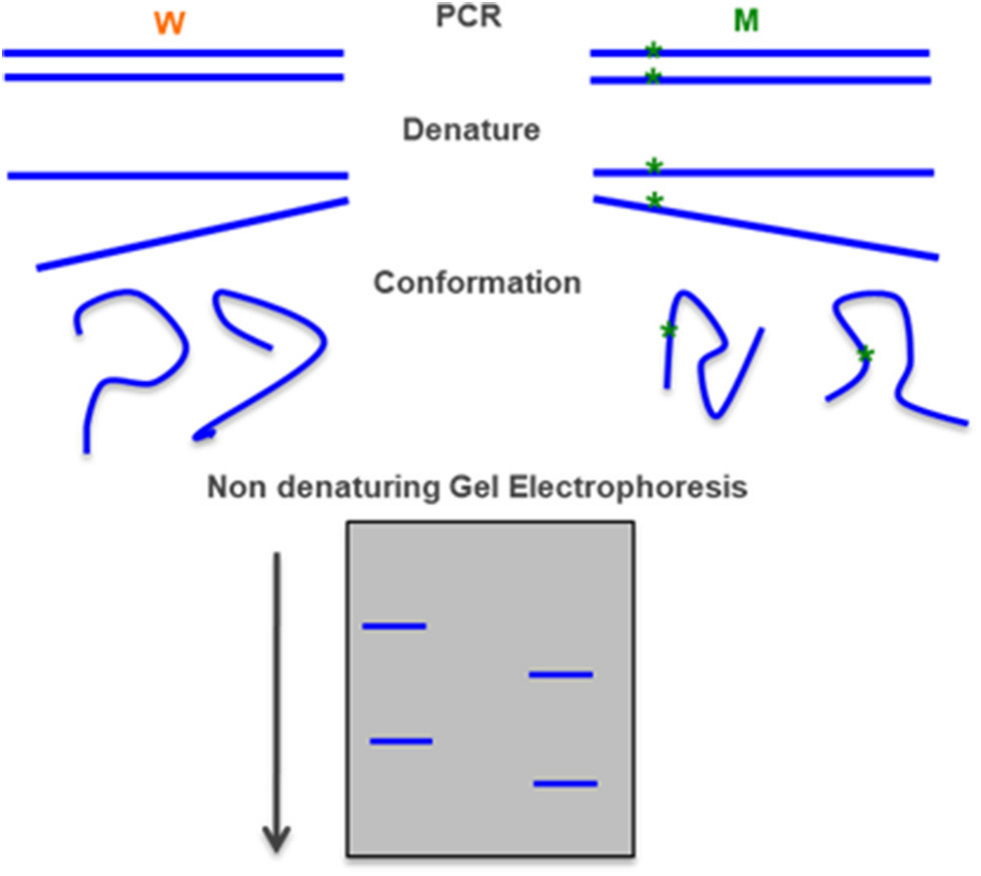
Single-stranded conformation polymorphism—a PCR-amplified DNA fragment is denatured in particular conditions and each the single-stranded fragment assumes a characteristic conformation that can be detected in a polyacrylamide gel electrophoresis. Adapted form Gasser et al. (91).

If double stranded DNA is denatured and then quickly returned to native conditions, it will remain in a single-stranded state with a characteristic conformation that can be detected by a change in the mobility of the segment during polyacrylamide gel electrophoresis under non-denaturing conditions. This method can detect novel pathogenic variant that would be missed by allele-specific approaches, but has some complexity in execution and interpretation (Figure 7) (74, 92).

### Allele-Specific PCR (ARMS)

**Figure 8:**
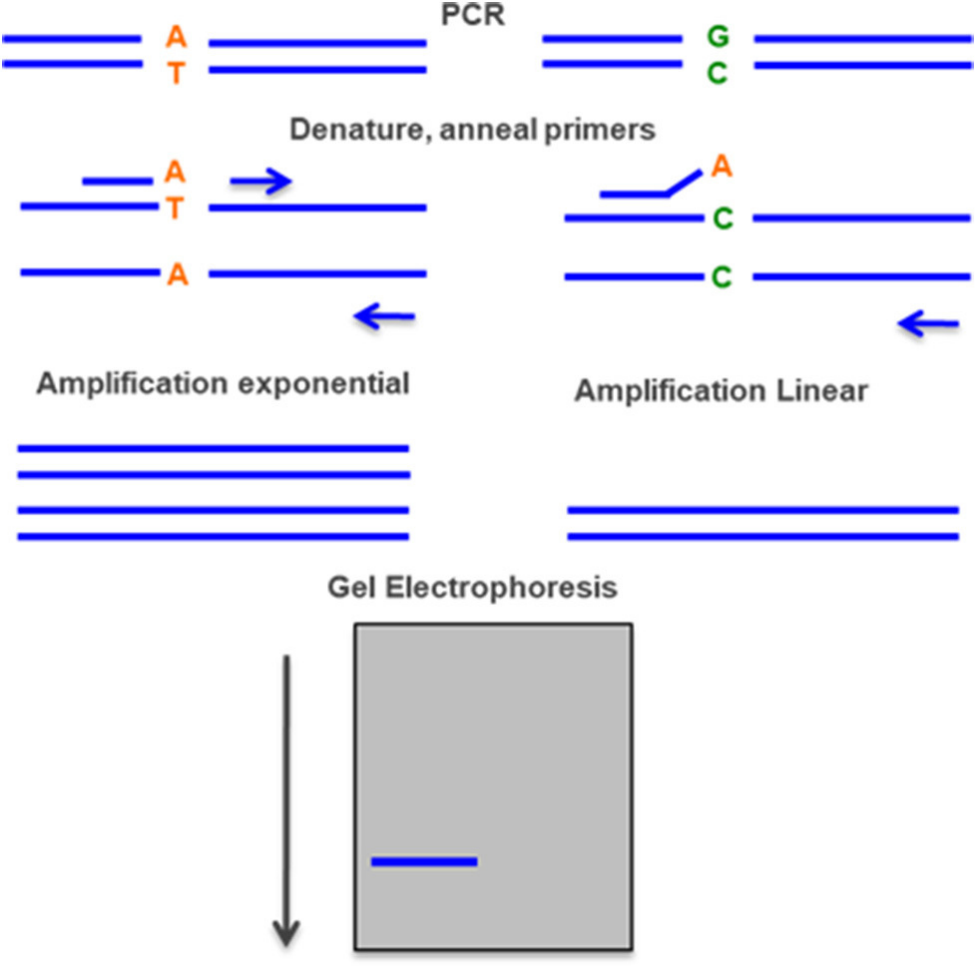
Allele-specific PCR—two PCR reactions are done simultaneously, with the same primer in one end and two different primers on the other end, one with the normal sequence and the other with the mutant. The rate of amplification is much higher with the specific primer and can be detected by gel electrophoresis.

In this method two alternative reactions are done for each pathogenic variant. Both PCR reactions use the same primer on one end, but at the other end each reaction uses a primer that corresponds to either the normal or mutant sequence. This technique has similar advantages to the amplification-created restriction site approach. The main differences are that it requires more PCR reactions but does not involve restriction digests (Figure 8) (93).

### Ligation Detection Reaction (LDR)

**Figure 9:**
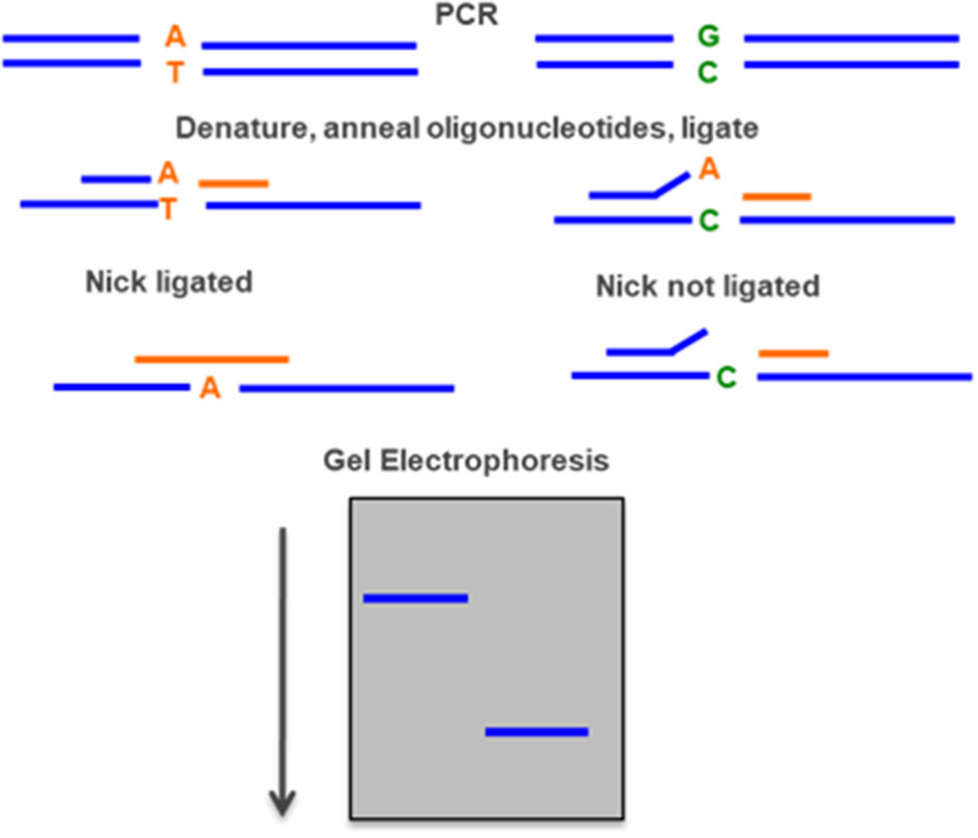
Ligase detection reaction—DNA ligase preferentially seal adjacent oligonucleotides hybridized to a DNA sequence when there is a perfect complementarity at the nick junction. A single base mismatch generates a different fragment detected by gel electrophoresis.

DNA ligase can discriminate point pathogenic variant by sequential rounds of linear template dependent ligation and preferentially seal adjacent oligonucleotides hybridized to target DNA in which there is perfect complementarity at the nick junction. A single base mismatch at the nick junction inhibits ligation and permits sequence discrimination at the single nucleotide level by the mobility on a sequencing gel. If the oligonucleotides are fluorescently labeled, the entire genotyping can be performed on an automated DNA sequencer (Figure 9) (94).

### D-HPLC

**Figure 10:**
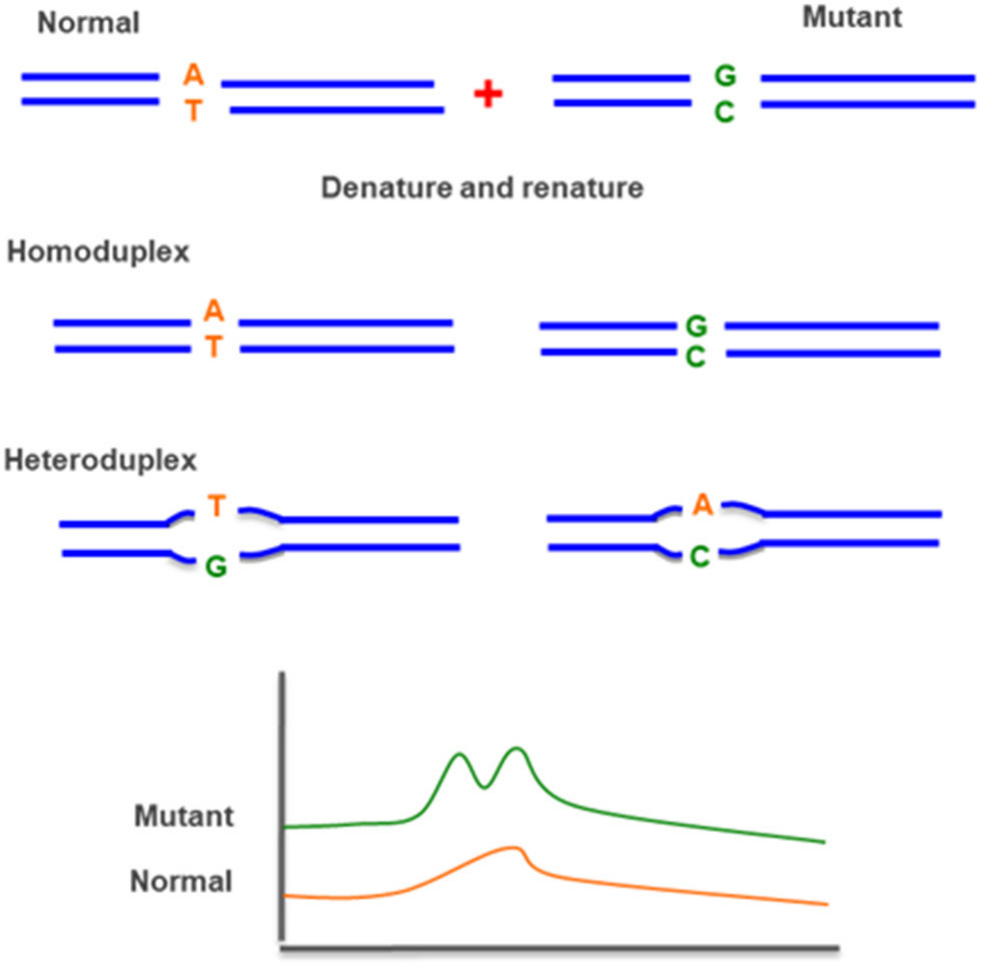
Denaturing high pressure liquid chromatography (D-HPLC)—uses heteroduplex formation between normal and mutated DNA strands. The different conformations are detected by ion-pair reverse-phase liquid chromatography.

Denaturing high pressure liquid chromatography (DHPLC) is a relatively new technique, which uses heteroduplex formation between wild-type and mutated DNA strands to identify pathogenic variant. Heteroduplex molecules are separated from homoduplex molecules by ion-pair, reverse-phase liquid chromatography on a special column matrix with partial heat denaturation of the DNA strands (Figure 10) (95, 96).

### Minisequencing and Multiplex Minisequencing

**Figure 11:**
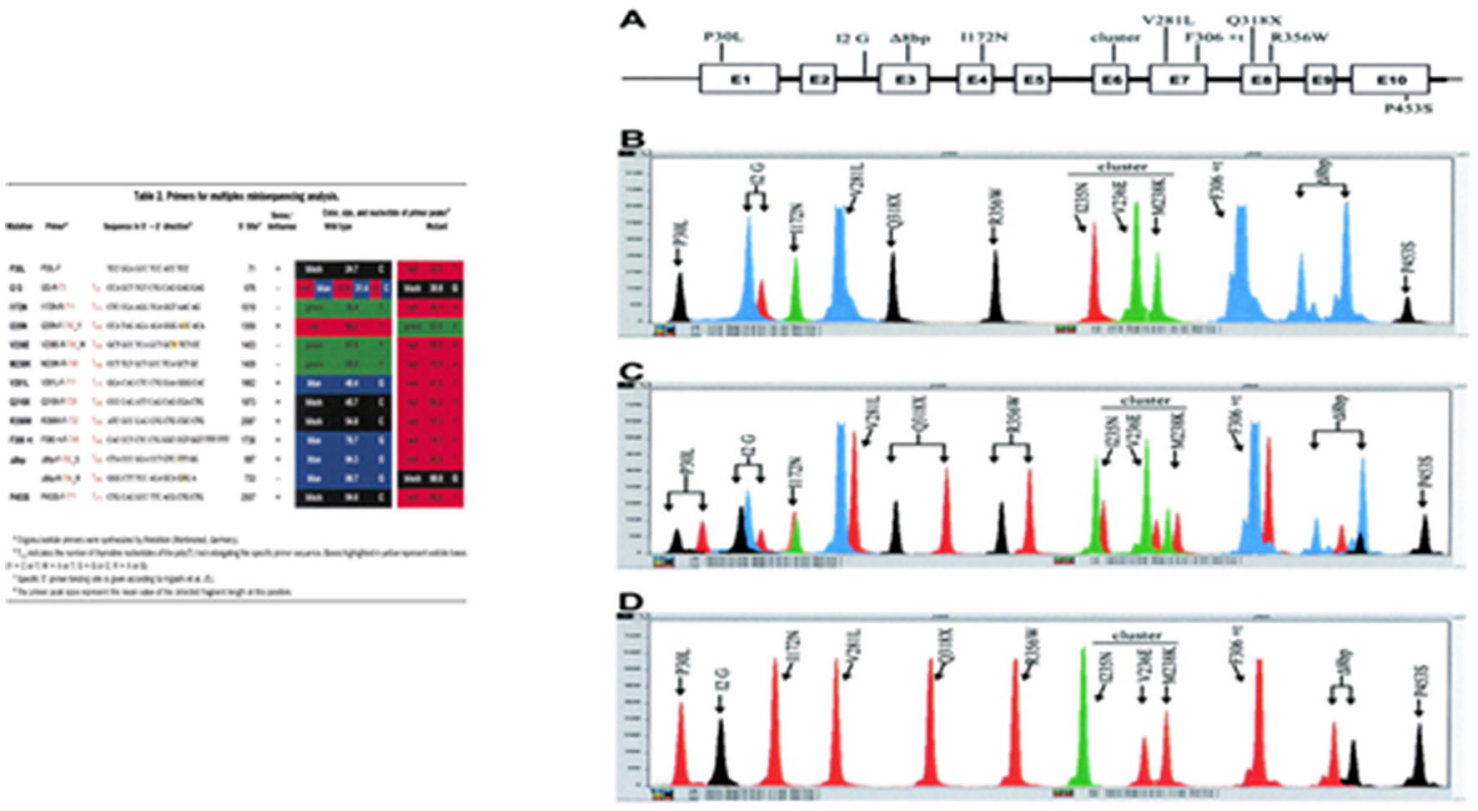
Minisequencing—a primer is hybridized to DNA next to a variant nucleotide site and extended with DNA polymerase by a single appropriate dideoxyribonucleotide triphosphate (ddNTP) that matches the nucleotide at the target site. A poly(T) sequence with different sizes is included in each primer at the 5′ end to facilitate the electrophoretic identification. Adapted from Krone et al. (97). **(A)** The CYP21 gene shown schematically with the nine most common mutations, transferred by apparent gene conversions from the CYP21P pseudogene. The P453S mutation is not present in the pseudogene, but occurs in 1–2% of mutant alleles. **(B)** CYP21 wild-type gene with heterozygosity for the A/C polymorphism at the intron 2 splice site (I2 G) position. **(C)** Mixture of CYP21 and CYP21P gene fragments demonstrating the detection of heterozygous mutations at every peak position. **(D)** CYP21P pseudogene amplicon with all common CYP21-inactivating mutations, demonstrating the detection of all mutations in a homozygous state.

In minisequencing, a primer is hybridized to DNA next to a variant nucleotide site and extended with DNA polymerase by a single appropriate dideoxyribonucleotide triphosphate (ddNTP) that matches the nucleotide at the target site. This method can be used in a multiplex reaction with primers elongated at the 5′ end with a poly(T) track of different sizes to facilitate electrophoretic separation of the diagnostic products (Figure 11) (97).

## Duplications and Deletions

A variety of methods are also available that can detect large deletions or duplications not only in the exonic or intronic regions of the CYP21A2 gene but also in the promoter and in contiguous regions as for the C4B gene.

### Southern-Blot

**Figure 12:**
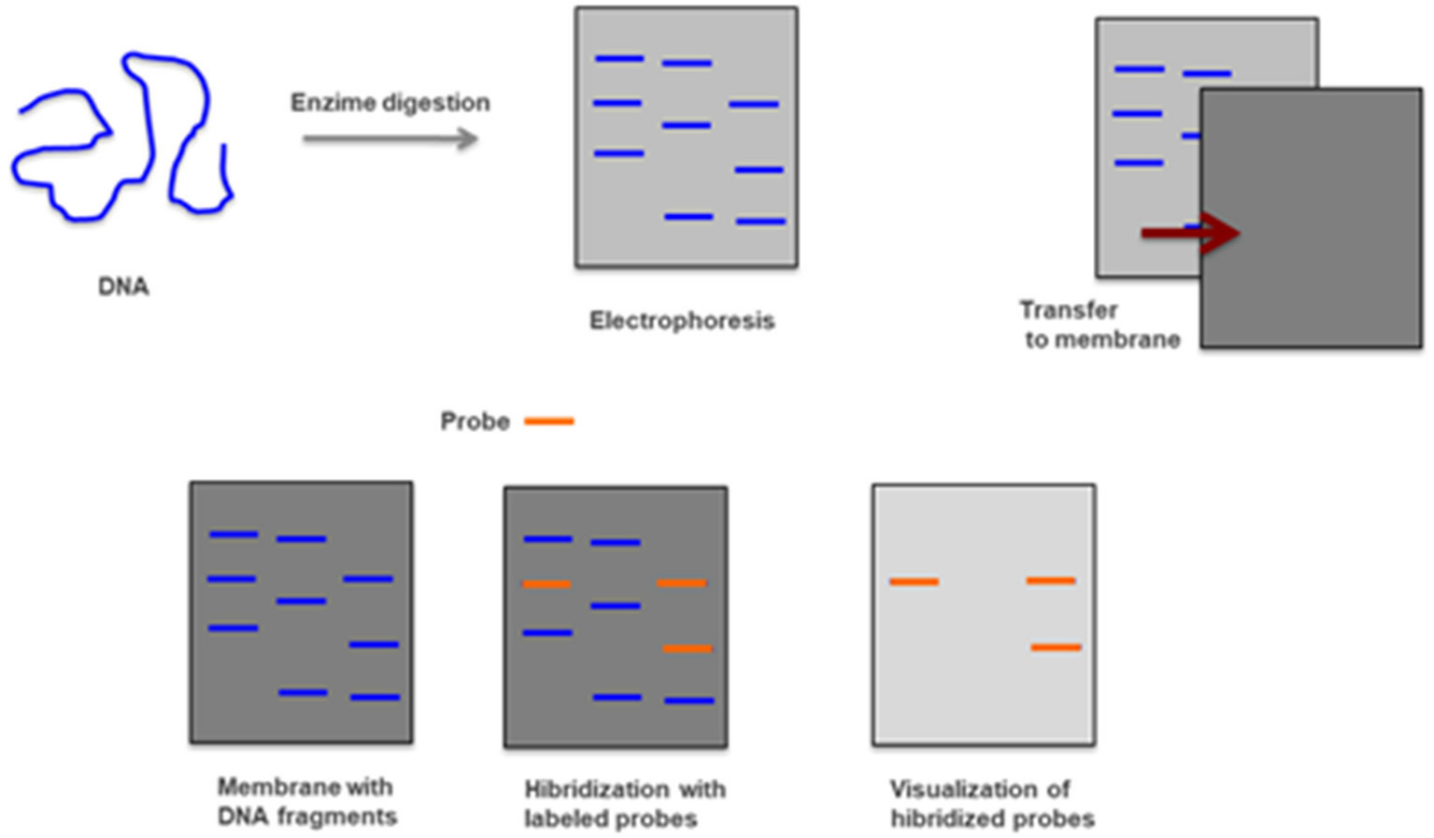
Southern-blot—Genomic DNA is digested with restriction enzymes and smaller fragments are obtained and separated by electrophoresis. After being transferred to a membrane and hybridized with radioactively labeled probes are detected by autoradiography.

This is a method that combines the transfer of electrophoresis and separation of DNA fragments to a filter membrane and subsequent fragment detection by probe hybridization. It usually uses genomic DNA, previously digested with restriction enzymes, to determine the number of sequences (e.g., gene copies) in a genome. Because it is a time-consuming and laborious method that uses radioactively labeled probes and requires a large amount of DNA it has been replaced by other techniques (Figure 12) (44, 98).

### Real Time PCR

**Figure 13:**
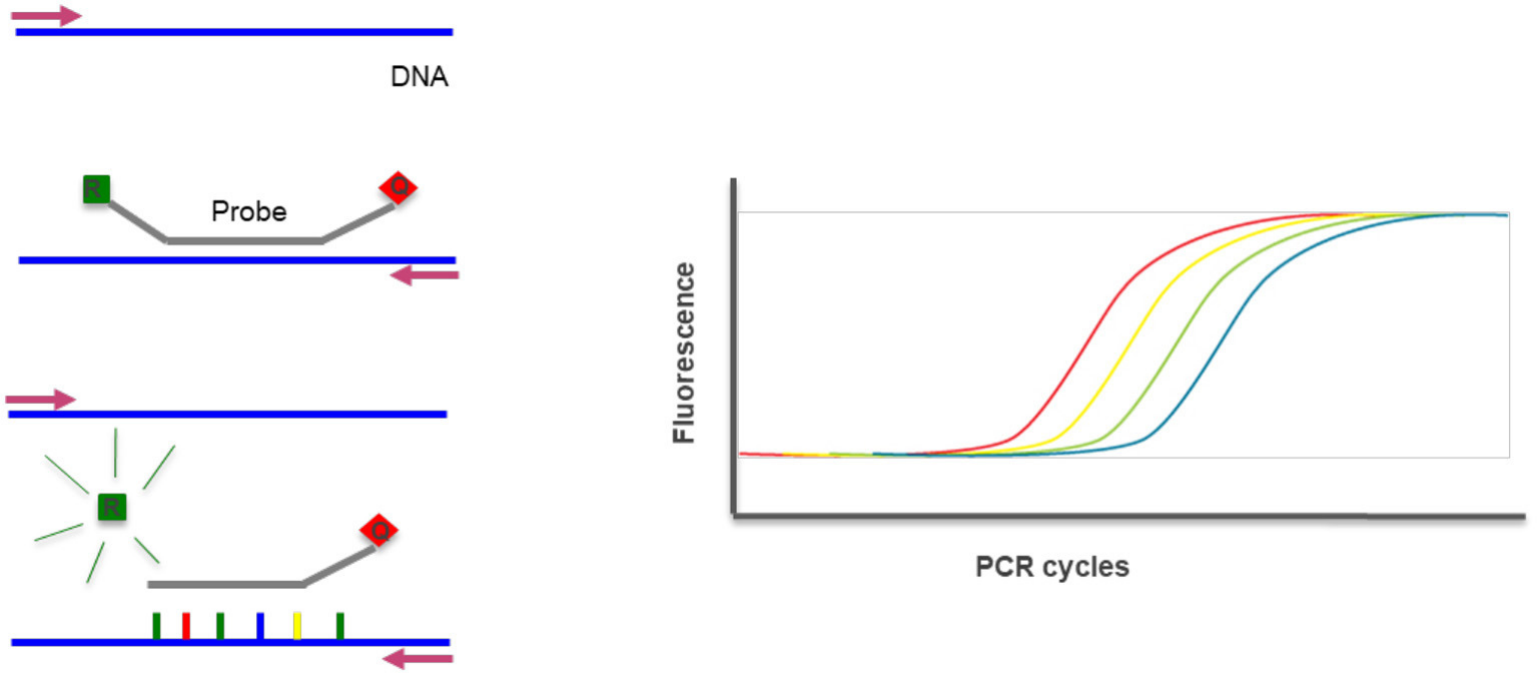
Real-time PCR—the method uses DNA probes with a fluorescent reporter at one end and quencher of fluorescence at the opposite end. The close proximity of the reporter to the quencher prevents detection of its fluorescence. During a PCR reaction, in each cycle the probe hibridyzes and elongates releasing the reporter that produces fluorescence detected and measured in real-time. The increase of fluorescence reflects the increase of product and can be quantified. R, Reporter; Q, Quencher.

Real time PCR is a technique where the progressions of a PCR reaction can be monitored in real time and simultaneously quantify the amount of product amplified. The method is based on the detection of the fluorescence produced by a reporter molecule which increases, as the reaction proceeds. This quantification can be used to assess gene copy number variations through a co-amplification of a control gene (Figure 13) (23).

### MLPA

**Figure 14:**
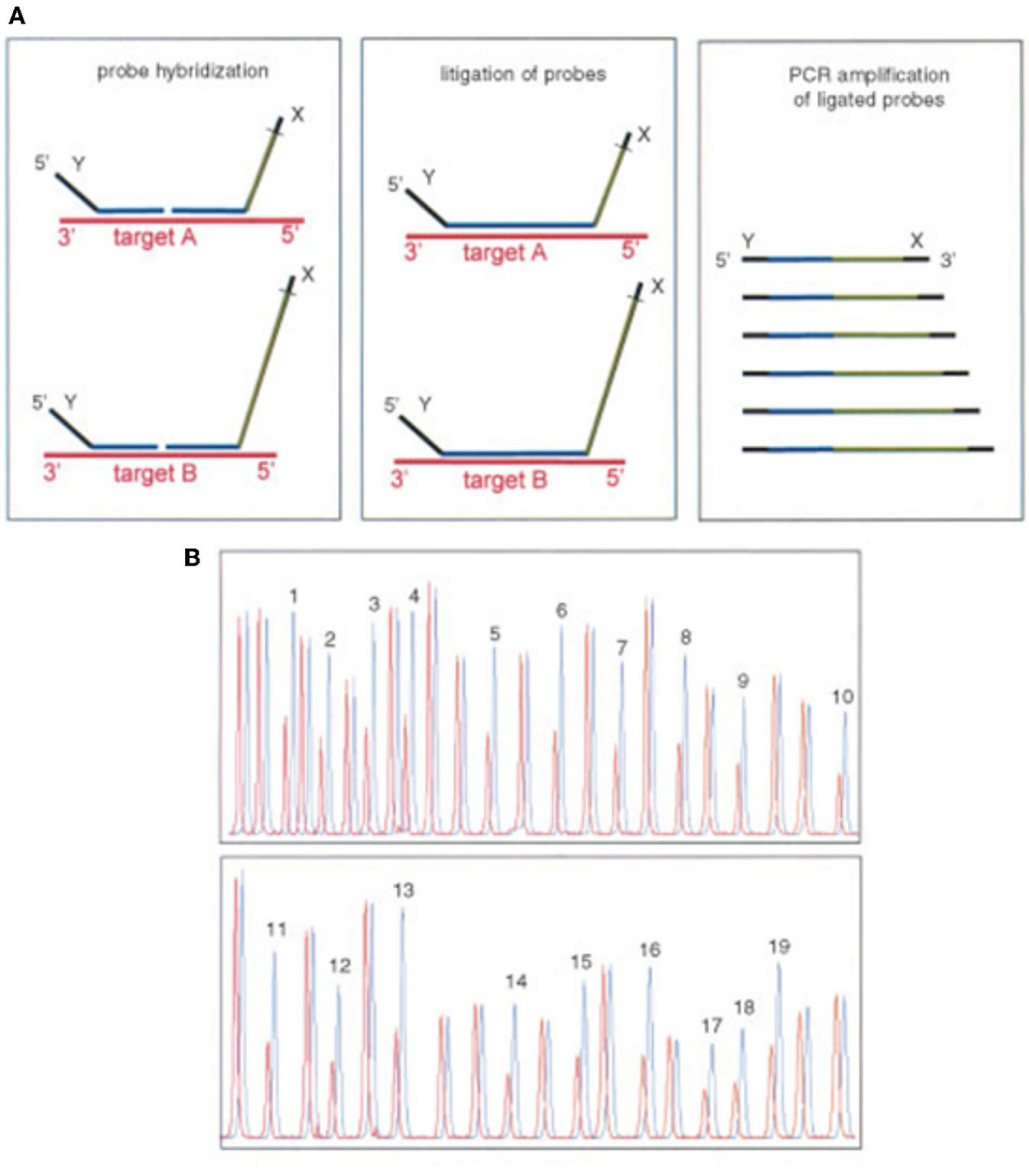
Multiplex ligation-dependent probe amplification (MLPA). Adapted from Schouten et al. (99). **(A)** Multiplex ligation-dependent probe amplification (MLPA) uses a single pair of primers and specific probes with progressive increasing lengths to be identified by electrophoresis. **(B)** Comparing the height of each peak of the sample with a control detects the number of copies.

Multiplex Ligation-dependent Probe Amplification (MLPA) assay is a technique that enables the detection of variations in the copy number of several human genes. Due to this ability, MLPA can be used for several molecular diagnosis of several different genetic diseases whose pathogenesis is related to the presence of deletions or duplications of specific genes. Moreover, MLPA assay can also be used in the molecular diagnosis of genetic diseases characterized by the presence of abnormal DNA methylation. Due to the large number of genes or genetic sequences that can be simultaneously analyzed by a single technique, MLPA assay represents the gold standard for molecular analysis of all pathologies derived from the presence of gene copy number variation. Detection of deletions and duplications of the *CYP21A2* gene and *CYP21A1P* pseudogene is currently performed by Multiplex Ligation—dependent Probe Amplification (MLPA), using the P050- CAH Kit (MRC-Holland). This high resolution method to detect copy number variation in genomic sequences uses only a single pair of PCR primers and the specificity relies on the use of progressively longer oligonucleotide probes in order to generate locus-specific amplicons of increasing size that can be resolved electrophoretically. Comparing the peak pattern obtained to that of the reference samples it is possible to determine which probes/locus show aberrant copy numbers (Figure 14) (99, 100).

## DNA Sanger Sequencing

**Figure 15:**
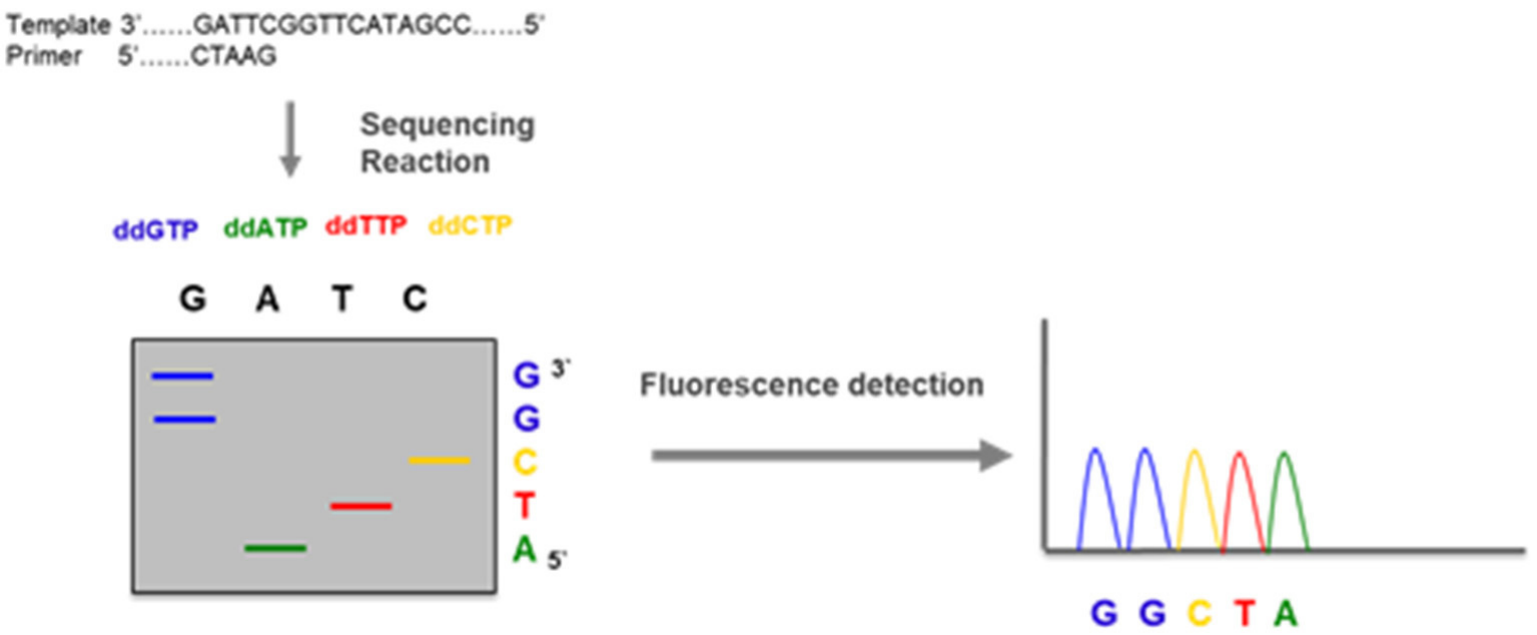
DNA sequencing—a DNA fragment amplified by PCR is used in an amplification reaction that, besides the normal nucleotides dATP, dGTP, dCTP, and dTTP, contains a mix of dye labeled terminator nucleotides (ddATP, ddGTP, ddCTP, and ddTTP). These modified nucleotides do not have the capacity of elongate the DNA chain and terminate the reaction when incorporated. The DNA sequence is obtained by electrophoresis that separates the fragments and fluorescence detection that identifies each of the nucleotides.

**Figure 16:**
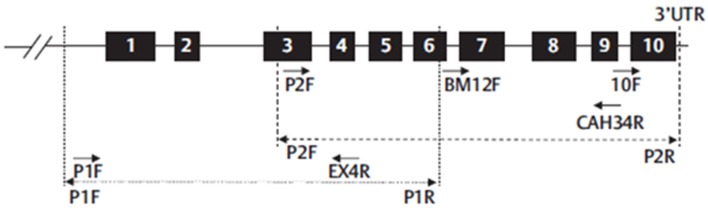
Schematic representation of *CYP21A2* gene structure and the strategy proposed to sequence whole gene. Numbered black boxes represent *CYP21A2* exons. Dot line (…) represent the first PCR-P1-amplification product (1,517 bp) and dash line (- - -) represent the second PCR-P2-amplification product (2,214 bp). Arrows represent the primers used in six different sequencing reactions (P1F and Exon4R for P1 fragment and P2F, BM12F, CAH34R, and 10 F for P2 fragment). Adapted from Carvalho et al. (102).

Nowadays, in many hospitals, a whole gene sequencing together with MLPA has become the standard procedures to genotype the CYP21A2 gene in cases of 21OH Deficiency. This is the method elected to detect pathogenic variants not screened by the targeted analysis and is also able to detect novel sequence variants. It usually covers the coding regions and the flanking intron-exon regions of the gene. *CYP21A2* whole genomic sequence may be performed selecting the functional *CYP21A2* gene and amplifying by PCR into 2 partially overlapping fragments, P1 and P2 (Figure 16), respectively with 1 517 and 2 214 base-pairs (bp), avoiding the co-amplification of the pseudogene *CYP21A1P* (101). After selective amplification of the targeted genes and subsequent purification, the PCR product is sequenced with internal primers that cover the entire *CYP21A2* gene (Figures 15, 16) (102).

## New Aspects in Genotyping

A final and promising aspect that can result from Genotyping is prevention. Preimplantation Genetic Diagnosis (PGD) can already be performed and be used to limit the transmission of the disease when used in conjunction with *in vitro* Fertilization (IVF).

For prenatal diagnosis the use of maternal circulating fetal DNA (Cff-DNA) allows early gender determination (*SRY*) in a precocious phase of pregnancy. This allows a timely identification of male fetuses that do not need to be treated prenatally contrarily to female fetuses in which doctors may want to prevent the occurrence of genital ambiguity. It is also possible to do sequencing of the *CYP21A2* gene in the fetal DNA circulating in maternal blood, but the technique is complex and still carries a significant possibility of false positive or false negative diagnoses. Chorionic villus sampling and amniocentesis still gives better outcomes but can only be performed rather late in view of the timing where genetic identification of Classic phenotypes of the disease would mostly benefit the decision process concerning the institution of dexamethasone suppressive treatment during the pregnancy (103).

## Conclusion

Congenital adrenal hyperplasia due to 21-hydroxylase deficiency are a group of very important diseases due to its high morbidity (the Classic forms) and its high prevalence (the Non-classic forms). They affect patient's life in many ways, going from salt wasting life-threatening crises to genital ambiguity with all its consequences of gender determination and reconstructive surgeries ultimately affecting normal sexuality and reproduction. This already highlights the importance of having a correct diagnosis to the level of a complete genetic characterization.

Perhaps more importantly than being infertile many of these patients often do not even attempt to conceive, but in those who wish to do it, genetic counseling is of particular importance.

The consequences of these diseases go still beyond, affecting growth and final height, body image, impacting on self-esteem and other psychological consequences including depression and anxiety. Attention to the consequences of overtreatment as well as under-treatment should always be present. In adults transitioning from pediatric care, adrenal crises and cardiovascular consequences together with the psychological well-being become the principal focus.

The diagnosis is first confirmed through 17OH-progesterone determinations which can be very high, moderately high or even normal at basal conditions needing confirmation through an ACTH stimulation test. The defining cut-off is generally considered to be between 10 and 15 ng/ml, either basally or post-ACTH. In the case of women these dosages should be done in the follicular phase of their menstrual cycles and in every patient the blood samples should be collected early in the morning. Newborn screening programs are very important as they permit the identification of severe cases at the ideal time for treatment thus being life-saving in many situations. In consequence of this screening programs, survival is no longer the major issue and has been replaced by the need to improve the patients' quality of life.

Suspected cases and even confirmed ones should be genotyped to completely characterize the pathogenic genetic variants. Both parents should also be analyzed to confirm that the pathogenic genetic variants affect both alleles. The actual recommendation involves the entire gene sequencing whenever that is possible. The main objectives are to confirm the diagnosis, delineate personalized therapeutic strategies and allow a correct genetic counseling.

## Author Contributions

DP and AP designed the paper. DP, BC, AP, AB, SG, and DM wrote and reviewed the article.

### Conflict of Interest Statement

The authors declare that the research was conducted in the absence of any commercial or financial relationships that could be construed as a potential conflict of interest.
