# Corrigendum: The Complexities in Genotyping of Congenital Adrenal Hyperplasia: 21-Hydroxylase Deficiency

**DOI:** 10.3389/fendo.2020.00113

**Published:** 2020-03-04

**Authors:** Duarte Pignatelli, Berta L. Carvalho, Aida Palmeiro, Alberto Barros, Susana G. Guerreiro, Djuro Macut

**Affiliations:** ^1^Hospital S. João, Porto, Portugal; ^2^Department of Biomedicine, Faculty of Medicine of Porto, Porto, Portugal; ^3^IPATIMUP/I3S Research Institute, University of Porto, Porto, Portugal; ^4^Genetics, Department of Pathology, Faculty of Medicine, University of Porto, Porto, Portugal; ^5^I3S Research Institute, University of Porto, Porto, Portugal; ^6^Clinical Genetics Center, Porto, Portugal; ^7^Clinic of Endocrinology, Diabetes and Metabolic Diseases, Faculty of Medicine, University of Belgrade, Belgrade, Serbia

**Keywords:** 21OH deficiency, CAH—congenital adrenal hyperplasia, adrenal cortex, androgen excess syndromes, genotyping, endocrine genetics, rare diseases, disorders of sex development

In the article, an author's name was incorrectly spelled as **Djuro Maçut**. The correct spelling is **Djuro Macut**. In addition there was an error in affiliation **7**. Instead of “**Department of Endocrinology, Diabetes and Metabolic Diseases, Faculty of Medicine, University of Belgrade, Belgrade, Serbia**,” it should be “**Clinic of Endocrinology, Diabetes and Metabolic Diseases, Faculty of Medicine, University of Belgrade, Belgrade, Serbia**.”

The authors apologize for this error and state that this does not change the scientific conclusions of the article in any way. The original article has been updated.

